# Addressing Practical Use of Viologen-Derivatives in Redox Flow Batteries through Molecular Engineering

**DOI:** 10.1021/acsmaterialslett.2c01105

**Published:** 2023-02-07

**Authors:** Rubén Rubio-Presa, Lara Lubián, Mario Borlaf, Edgar Ventosa, Roberto Sanz

**Affiliations:** † Department of Chemistry, University of Burgos, Plaza Misael Bañuelos s/n, E-09001 Burgos, Spain; ‡ International Research Centre in Critical Raw Materials-ICCRAM, University of Burgos, Plaza Misael Bañuelos s/n, E-09001 Burgos, Spain

## Abstract

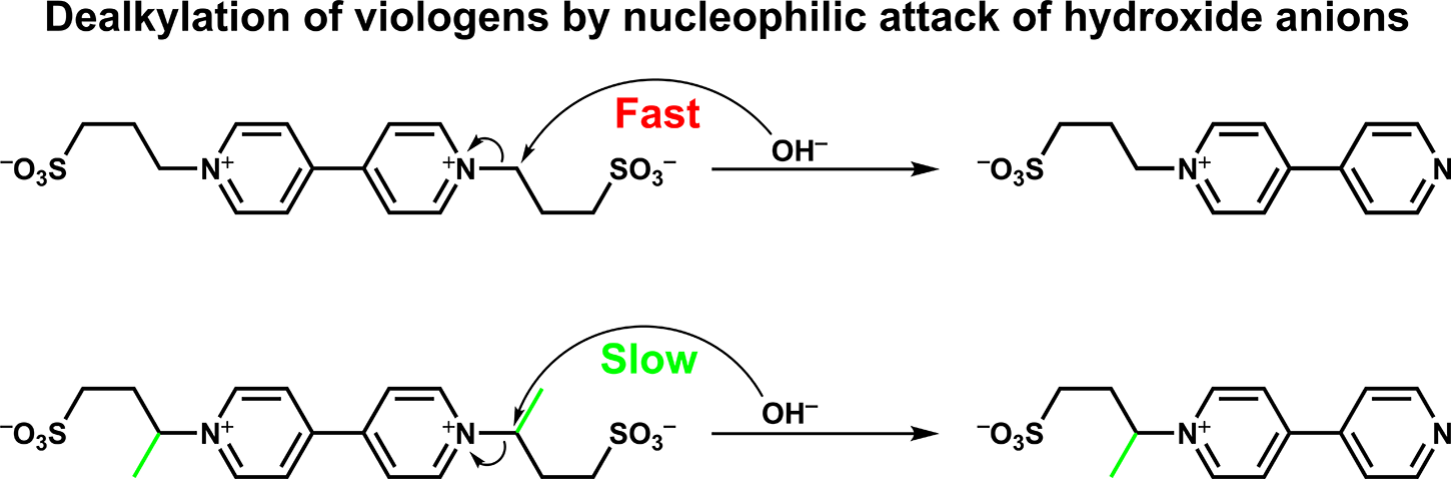

In practical scenarios, viologen-derivatives face an accelerated degradation in the unavoidable presence of traces of oxygen in large-scale redox flow batteries. Herein, we confirm the primary degradation mechanism and propose a straightforward, cheap, and fast method to evaluate the stability of viologen-derivatives toward this degradation. Considering that the cleavage of the N-substituent is the main proposed pathway for viologen degradation, a new viologen-derivative, bearing an alkylsulfonate chain with a secondary carbon center joined to the N atom, is synthesized to illustrate how molecular engineering can be used to improve stability.

Generation of energy from renewable sources such as wind and solar radiation has become a key priority for our society. Unfortunately, the unpredictable intermittency of these sources requires energy storage to match energy production and demand. Among the various energy storage systems, redox flow batteries are called to play a key role in the transition toward a sustainable and environmentally friendly energy system. Featured by its independent scalability of energy and power, the redox flow battery is a promising alternative for stationary energy storage. The all-vanadium flow battery is the most mature redox flow battery technology.^1^ However, vanadium is considered a critical raw material for the United States and European Union, which has triggered the interest to replace vanadium species with more sustainable and abundant active species.^1^ Aqueous organic redox flow batteries (AORFBs) are bringing much attention, since active species are organic and organometallic molecules based on Earth-abundant elements. So far, many organic molecules such as anthraquinone-derivatives,^2^ phenazine-derivatives,^3^ or fluorenone-derivatives^4^ have been shown to deliver high performance in alkaline media. At neutral pH, viologen-derivatives are the most commonly used active species for anolytes.^1^ Their performances in terms of energy density and cycle stability have been improved in recent years by modification of the basic structures.^5,6^ The 1,1′-bis[3-sulfonatopropyl]-4,4′-bipyridinium (BSPr-Vi) is one of the most commonly used viologen-derivatives in the literature, achieving high cycle stability, which is a key parameter for stationary energy storage, at laboratory scale.^7^ One of the main differences for viologen-derivatives at laboratory scale is the extremely protected atmosphere for the anolyte, as the best cyclability performance is achieved using an Ar-filled glovebox.^7−9^

The presence of oxygen dissolved in the electrolyte needs to be avoided, especially for the negative comportment. The irreversible reduction of oxygen, either through electrochemical reaction in the electrodes or spontaneous charge transfer with reduced active species in the anolyte, leads to the irreversible consumption of charges during the charge process. As a result, the anolyte does not reach a full state of charge at the end of the charge process, limiting the discharge capacity and shorting the energy storage capacity. Importantly, the presence of oxygen leads to faradaic imbalance between the anolyte and catholyte, since the fully charged catholyte cannot be fully discharged, resulting in a progressive loss in charge capacity upon cycling.^10^ This capacity fading is not related to degradation of active species but faradaic imbalance. For research purposes, this issue is overcome by carrying out the measurements inside an Ar-filled glovebox in the complete absence of oxygen.^7−9^ Alternatively, faradaic imbalance can be addressed for academic purposes by oversizing the positive reservoir so that a large excess of electroactive species is added to the positive compartment.^4,9,11^ While these approaches are very useful to study the intrinsic properties at the research level, complete absence of oxygen for long periods (>15 years) at large scale (>MWh) will be very challenging, if not impossible, to be achieved. Fortunately, several strategies have been recently proposed to amend this issue through various faradaic rebalance approaches.^12−14^ These strategies have been successfully implemented in all-vanadium flow batteries as well as aqueous organic flow batteries in alkaline media at laboratory scale. However, they have not been implemented in AORFBs in neutral pH. Since these scalable strategies are not based on complete exclusion of air, one needs to assess the stability of the state-of-the-art viologen-derivatives in a redox flow battery having an oversized positive compartment so that the intrinsic stability of the viologen-derivatives under “standard exclusion of air” needs to be determined. Figure 1A reveals a significant capacity fading for a system in which the negative compartment (BSPr-Vi) was purged with Ar and kept under Ar overpressure, referred to as *standard air exclusion*, and an excess of potassium ferrocyanide was added to the positive compartment. In contrast to these results, reports in the literature that were carried out inside an Ar-filled glovebox show higher capacity retention values.^7−9^ This indicates that the presence of small amounts of oxygen remaining in the anolyte for our experiment triggers an accelerated degradation of this state-of-the-art viologen-derivative. To confirm this, the negative compartment was briefly exposed to air after being operated for 40 cycles under Ar overpressure (Figure 1B). While the excess of ferrocyanide should be able to compensate the irreversible charges consumed by the reduction of oxygen (ca. 40% of the charge capacity according to the Coulombic efficiency), an irreversible and drastic drop in charge capacity was observed onward from cycle 40. This behavior confirms that the presence of oxygen in the anolyte leads to accelerated degradation of viologen-derivatives. Among the various degradation mechanisms proposed in the literature,^15^ dealkylation through a nucleophilic attack of hydroxide anions (OH^–^) giving rise to the C(alkyl)–N bond cleavage, which was initially suggested by Bird and Kuhn^16^ as well as Rieger and Edwards^17^ and recently revisited by Aziz’s group,^18^ gained our attention. It should be noted that the reduction of oxygen generates hydroxide anions, increasing the pH value of the electrolyte (O_2_ + 2 H_2_O + 4 e^–^ → 4 OH^–^).

**Figure 1:**
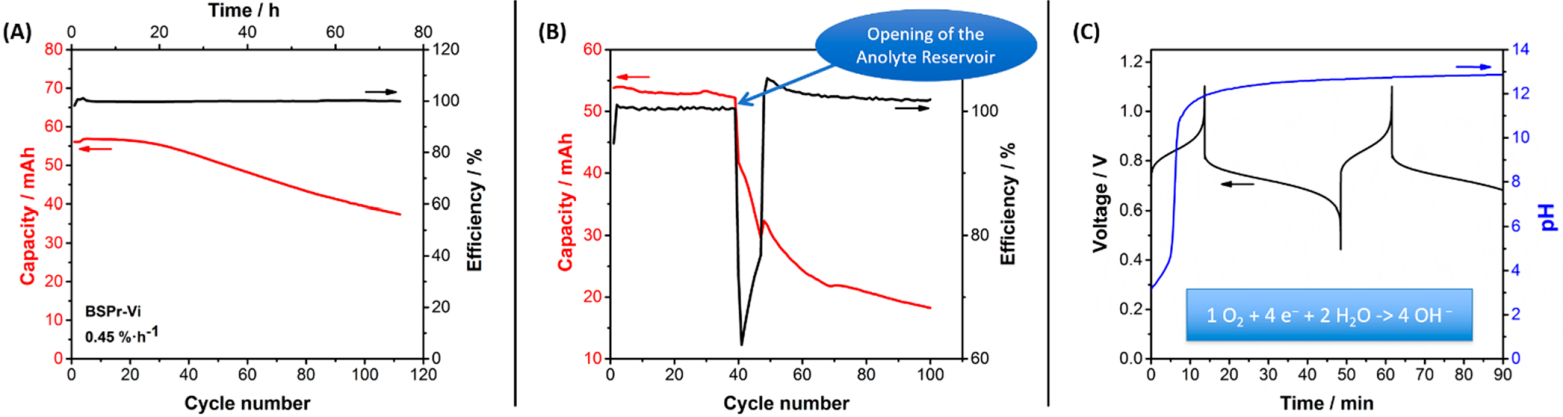
(A) Evolution of the charge capacity and Coulombic efficiency of the cell upon cycling having BSPr-Vi//K_4_[Fe(CN)_6_] in 1 M KCl using Ar overpressure in the negative compartment. (B) Evolution of the charge capacity and Coulombic efficiency of the cell upon cycling having BSPr-Vi//K_4_[Fe(CN)_6_] in 1 M KCl using Ar overpressure in the negative compartment. The negative reservoir was temporarily open in cycle no. 40. (C) Evolution of the cell voltage and the pH value in the anolyte during the first two cycles.

Thus, a pH probe was immersed in the negative reservoir to monitor the evolution of the pH value during the first few cycles. The results (Figure 1C) clearly show that the pH value increased during the first charge process. Note that a small delay in pH monitoring is likely due to the large size of the pH probe. The changes in pH value can be estimated theoretically by knowing the irreversible charges consumed in a cycle and the Faraday constant (Section S1). For instance, a Coulombic efficiency in the first cycle of 98%, which is a very good value without a glovebox, results in a pH change from 4 to 12 due to the logarithmic scale of pH values. Likewise, four cycles at 99.5% due to a very small temporary leakage of air would have the same impact in the pH value. This means that any electroactive species that are designed to be implemented in neutral pH at large scale should withstand mild alkaline media, since the unavoidable entry of oxygen will change pH values to mild alkaline values. Even if the pH value is corrected by, e.g., intentionally evolving oxygen in the positive compartment, the electroactive molecule will be temporarily exposed to mild alkaline values at some point of its expected long cycle life. While a pH buffer can temporally maintain the pH value, it will require the addition of chemicals in the electrolyte, which will be eventually depleted.

Fortunately, one major advantage of organic electroactive species is the tunability of their properties through molecular engineering. This feature could be potentially used to improve the stability of viologen-derivatives against mild alkaline values for enhancing the practical uses of this family of molecules. First, the degradation mechanism was further investigated. Since the mono-*N*-alkylated 4,4′-bipyridine derivative, 3-([4,4′-bipyridin]-1-ium-1-yl)propane-1-sulfonate (MSPr), should be a degradation product derived from the nucleophilic attack of hydroxide anions, this compound was independently synthesized and characterized. NMR analysis of the anolyte after 3 days of cycling under Ar overpressure revealed that there is a correlation between the capacity fading and the presence of MSPr in the NMR spectra (Figure 2), which confirms that the proposed degradation mechanism by dealkylation is likely the main contributor. Indeed, the presence of ca. 35% of the electrochemically inactive mono-*N*-alkylated 4,4′-bipyridine MSPr (NMR spectrum, Figure 2) relates well to the capacity fading of 35% after 3 days of cycling (capacity retention, Figure 2). In terms of molecular engineering, the addition of an alkyl group at the α-position of the *N*-substituent could hinder the attack by stereoelectronic effects and, therefore, increase the stability of the viologen-derivative in mild alkaline conditions. An efficient synthetic route was developed to synthesize a new viologen, 3,3′-([4,4′-bipyridine]-1,1′-diium-1,1′-diyl)bis(butane-1-sulfonate) (BS3Bu-Vi), as displayed in Scheme 1. BS3Bu-Vi was accessed from 4,4′-bipyridine by its double alkylation with 1,3-butane sultone, which in turn was obtained using a previously described method involving double mesylation of 1,2-propanediol followed by α-lithiation to the S atom and subsequent intramolecular nucleophilic displacement of the primary mesylate.^19^ The detailed synthetic procedures are described in Section S3. Gratifyingly, cyclic voltammetry (Figure S1, Section S2) of the new viologen (BS3Bu-Vi) presents similar electrochemical behavior to that of the state-of-the-art viologen (BSPr-Vi).

**Figure 2:**
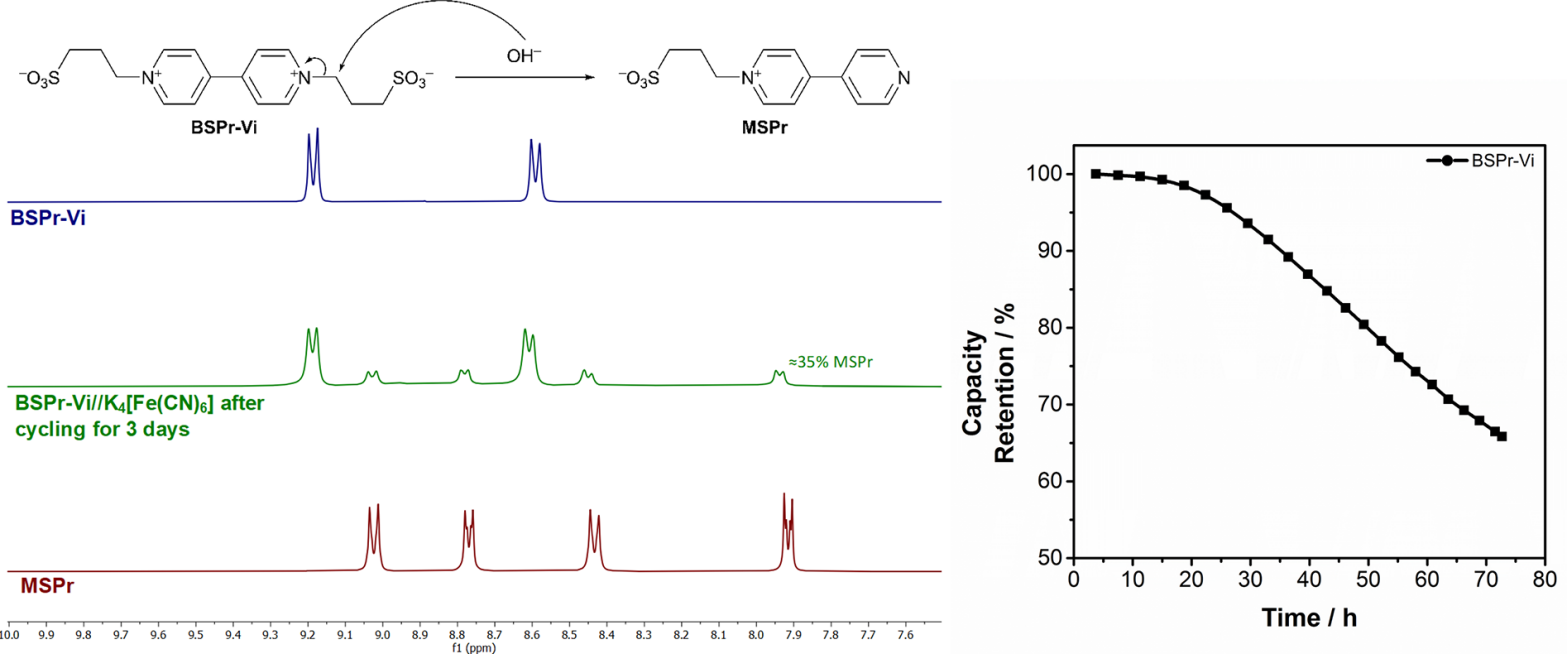
^1^H NMR postmortem analysis of the BSPr-Vi//K_4_[Fe(CN)_6_] battery anolyte after 3 days, and capacity fading of the BSPr-Vi//K_4_[Fe(CN)_6_] battery over 3 days.

**Scheme 1:**
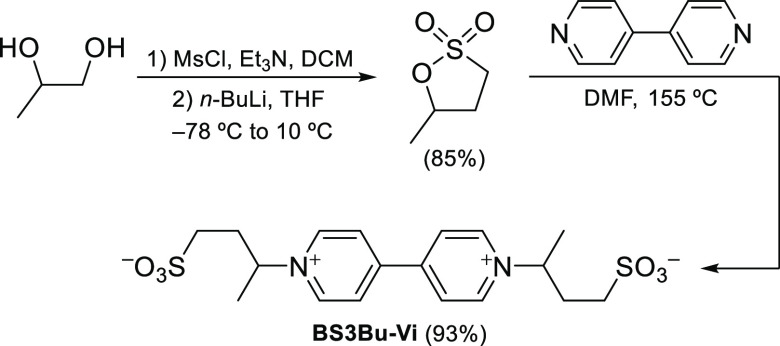
Synthesis of BS3Bu-Vi

Since the degradation mechanism requires the consumption of one hydroxide anion per molecule of viologen-derivative (equation in Figure 2), its degradation could be monitored by recording the evaluation of the pH value with time starting at a mild alkaline value in a beaker (the hydroxide anion should be consumed during degradation of viologen). Figure 3A shows the evolution of the pH value starting from pH = 11 for two viologen-derivatives: BSPr-Vi (state-of-the-art) and BS3Bu-Vi (new viologen-derivative). Two main conclusions are drawn. First, the pH value indeed decreases over time. Second, a slower change in pH values with time is observed for the new viologen-derivative compared to the state-of-the-art, which suggests a higher stability against this type of degradation mechanism. Note that the amount of degraded product generated in this test is not sufficient for unambiguous quantitative NMR analysis. Redox flow batteries were then assembled and evaluated to explore the benefits of molecular design for viologen-derivatives in their cycle stability. It should be noted that the capacity fade rate is reported against time as a more comparable metric to assess electrolyte lifetime.^20^ For a fair comparison, both battery cells were evaluated for 3 days (72 h), and their capacity fading (% h^–1^) was calculated as the total capacity loss after 3 days normalized by 72 h (Figures 1A and 3B). The capacity fading of 0.45 and 0.15% h^–1^ was obtained for the BSPr-Vi and the new viologen (BS3Bu-Vi), respectively, confirming the higher stability of the latter and the ability to engineer viologen-derivatives for improving their stability (Figure S2, Section S3). It should be noted that Coulombic efficiencies in both cases were rather high considering that glovebox was not used: ca. 98% in the first cycle and >99.9% after a few cycles. As mentioned before, the 2% Coulombic inefficiency in the first cycle would translate in a change in pH from 4 to 12. Indeed, the pH value of the electrolyte was measured after cycling for 3 days, and values above 10 were obtained. It should be noted that crossover of viologen through the membrane did not contribute significantly to the capacity fading as revealed by the postmortem cyclic voltammetries of the catholytes after cycling (Figure S3, Section S4). NMR postmortem analysis of the anolyte after cycling revealed the presence of mono-*N*-alkylated 4,4′-bipyridine (MS3Bu), confirming the dealkylation as the main degradation mechanism for the new viologen as well (Figure S4, Section S5).

**Figure 3:**
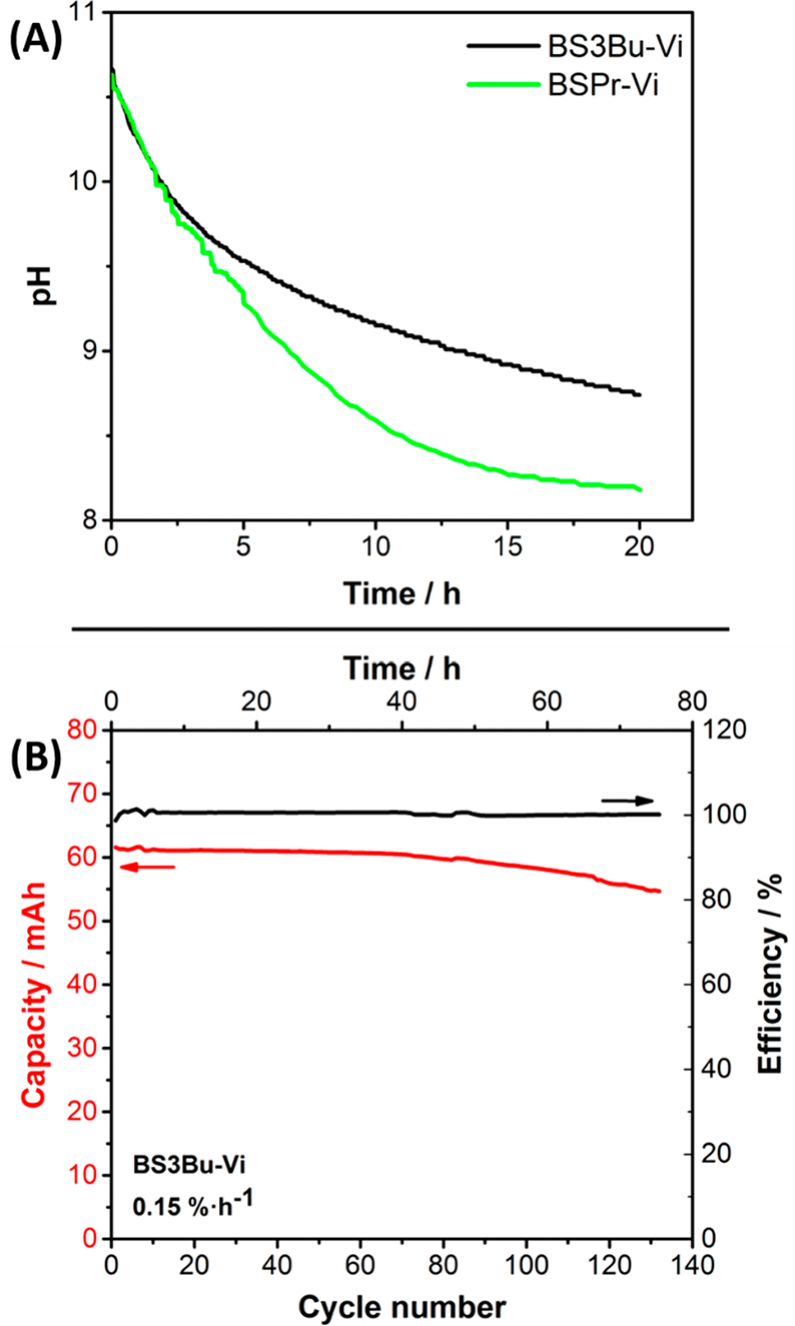
(A) Evolution of the pH value over 20 h for the BSPr-Vi and the BS3Bu-Vi. (B) Evolution of the charge capacity upon cycling over 72 h for redox flow battery cells using the BS3Bu-Vi.

In conclusion, the generation of hydroxide anions from the reduction of oxygen present in the anolyte of viologen-based redox flow batteries is confirmed to accelerate their degradation via dealkylation through nucleophilic attack, leading to a decrease in capacity retention. Thus, the new generation of viologen-derivatives should withstand mild alkaline media for being deployed at large-scale under realistic conditions, as the entry of small amounts of oxygen in large flowing devices is very challenging to prevent. Herein, it was shown that monitoring of the pH starting from mild alkaline media is a fast, cheap, and direct method to evaluate the chemical stability against dealkylation by nucleophilic attack. In addition, a new viologen-derivative was proposed to demonstrate that stability can be increased through molecular engineering. The introduction of a methyl group in the alkyl chain of the viologen at the α-position with respect to the N atoms resulted in higher stability, which is attributed to a greater difficulty for the nucleophilic attack of the hydroxide. Because this one major challenge for practical uses of viologen-derivatives was identified, a simple method for evaluation was proposed, and a new viologen-derivative was synthesized for probing the suitability of molecular engineering to address this issue, which clearly illustrates that molecular engineering will play a key role in progressing toward practical deployment of viologens.

## Supporting Information Available

General methods, theoretical estimation of the changes in pH, synthesis and characterization of all compounds, capacity fading and comparative capacity retention, crossover evaluation, and NMR postmortem analysis (PDF)
