# Supplementary material for: Addressing Practical Use of Viologen-Derivatives in Redox Flow Batteries through Molecular Engineering
Source: ACS Mater Lett. 2023 Feb 7;5(3):798–802. doi: 10.1021/acsmaterialslett.2c01105 (PMC9993555; doi:10.1021/acsmaterialslett.2c01105)
Supplement: Supplementary file 1 — tz2c01105_si_001.pdf [file tz2c01105_si_001.pdf]

Addressing Practical Use of Viologen-Derivatives in Redox Flow Batteries through Molecular Engineering

Rubén Rubio-Presa[1], Lara Lubián,[1,2] Mario Borlaf,[1,2] Edgar Ventosa[1,2,*], and Roberto Sanz[1,*]

[1]*Departamento de Química, Facultad de Ciencias, Universidad de Burgos, Pza. Misael Bañuelos s/n, E-09001-Burgos, Spain.*

[2]*International Research Centre in Critical Raw Materials-ICCRAM, University of Burgos, Plaza Misael Bañuelos s/n, E-09001, Burgos, Spain.*

Electronic Supplementary Information

Index

General methods S2

Section S1. Theoretical estimation of the changes in pH value S4

Section S2. Synthesis and characterization S6

Section S3. Comparative capacity retention S18

Section S4. Anolytes crossover evaluation S18

Section S5. Capacity fading and NMR post mortem analysis for **BS3Bu-Vi** S19

References S20

General methods

Materials.

All common reagents and solvents were purchased from Aldrich or Alfa-Aesar and used as received without further purification.

NMR measurements.

NMR spectra were measured on Bruker Avance 300 MHz spectrometer. ^{1}H NMR: splitting pattern abbreviations are: s, singlet; d, doublet; t, triplet; q, quartet; dd, double doublet; ddd, doublets of doublets of doublets; ddt, double doublet of triplets; dt, doublet of triplets; dq, doublet of quartets; td, triplet of doublets; qd, quartet of doublets; p, pentuplet; h, sextet; hept, heptet; m, multiplet; br, broad; a, apparent; the chemical shifts are reported in ppm using residual solvent peak as reference. ^{13}C NMR spectra were recorded at 75.4 MHz or 100.6 MHz using broadband proton decoupling and chemical shifts are reported in ppm using adequate solvent peaks as internal reference ($(CH_3)_2CO$: 30.89; CH_3OH: 49.50) and the multiplicities were determined by DEPT experiments.

Low resolution mass spectra.

Low resolution mass spectra (LRMS) measurements were recorded on an Agilent 6890N/5973 Network GC System, equipped with a HP-5MS column using electronic impact (EI).

High resolution mass spectra.

High resolution mass spectra (HRMS) were recorded with a LC-MS system equipped with an Agilent 1260 Infinity Liquid Chromatography instrument and an Agilent 6545 Q-TOF Mass Spectrometer using ESI ion sources, as specified.

Melting points determination.

Melting points were measured on a Gallenkamp apparatus using open capillary tubes and are uncorrected.

pH measurement.

pH measurements were determined using an Accument AB150 device which allows to record pH values at different times.

Cyclic voltammetries.

Cyclic voltammetry studies were performed using an Autolab PGSTAT12 (Methrom-Autolab, The Netherlands) with NOVA 2.1.3 software. A three-electrode cell was employed using a polished glassy carbon working electrode ($A_{electrode}$ = 7 mm^2), a Pt wire counter electrode (99% purity) and the Ag/AgCl (3 M KCl) electrode was used as aqueous reference electrode.

Flow batteries.

Filter-pressed flow cells using Nafion 212 and graphite felt as the ion selective membrane and electrodes were used in this study. The projected area of the cell was 9 cm^2. The flow rate was fixed at ca. 50 $mL·min^{-1}$. Galvanostatic charge-discharge measurements were performed using a Neware BTS battery testing system CT-40087-5V6A-S1. The batteries were charged at 30 $mA·cm^{-2}$ with voltage limits at 1.1 V. Thereafter, batteries were discharged between −20 and −10 $mA·cm^{-2}$ with a voltage limit of 0.45 V under Ar atmosphere. General conditions: anolyte (13 mL), 0.2 M viologen in 1.0 M KCl; catholyte (45 mL), 0.3 M $K_4[Fe(CN)_6]$ in 1.0 M KCl.

Section S1. Theoretical estimation of the changes in pH value

The reduction of oxygen present in the anolyte generates hydroxide anions turning the electrolyte more alkaline.

$$O_2 + 4\ H_2O + e^- \rightarrow 4\ OH^-$$

The change in pH can be theoretically estimated from the coulombic efficiency assuming that all the irreversible charges are spent in the reduction of oxygen. The **Equation S1** correlate the irreversible changes with the concentration of oxygen through the Faraday constant equation and the stoichiometry reaction.

$$Q_i = [O_2] \cdot F \cdot e^-$$

Equation S1

Where Q_i is the irreversible capacity, F is the Faraday constant (mAh/mmol) and V is the volume of the sample (mL).

The following **Equation S2** can be obtained through the **Equation S1** to calculate the change in pH at the end of the first cycle as a function of the irreversible charge (coulombic inefficiency).

$$pH = 14 + \left[\log \frac{Q_i}{F \cdot V}\right]$$

Equation S2

Table S1 shows the relation between the coulombic efficiency and the final pH of the anolyte.

Furthermore, it can be seen that a minimum amount of oxygen, as an irreversible capacity of 1%, generates a sufficient concentration of OH^- to provide a pH of 11.30.

As the irreversible charge increases, a higher amount of OH^- is generated leading to a more alkaline pH value.

Table S1. Battery pH calculation as a function of initial cell oxygen.

pH_0	[Vi] (M)	Volume (mL)	C.E. (%)	Q_i (mAh)	Mole OH^-	pOH_F	pH_F
**4.00**	0.20	13.00	100.00	0.00	0.00	10.00	4.00
**4.00**	0.20	13.00	99.00	0.70	$2.60 \cdot 10^{-5}$	1.47	11.30
**4.00**	0.20	13.00	98.00	1.41	$5.25 \cdot 10^{-5}$	1.17	11.61
**4.00**	0.20	13.00	97.00	2.13	$7.96 \cdot 10^{-5}$	0.99	11.79
**4.00**	0.20	13.00	96.00	2.87	$1.07 \cdot 10^{-4}$	0.86	11.92
**4.00**	0.20	13.00	95.00	3.63	$1.35 \cdot 10^{-4}$	0.75	12.02
**4.00**	0.20	13.00	94.00	4.40	$1.64 \cdot 10^{-4}$	0.67	12.10
**4.00**	0.20	13.00	93.00	5.19	$1.93 \cdot 10^{-4}$	0.60	12.17
**4.00**	0.20	13.00	92.00	5.99	$2.23 \cdot 10^{-4}$	0.54	12.24
**4.00**	0.20	13.00	91.00	6.81	$2.54 \cdot 10^{-4}$	0.48	12.29
**4.00**	0.20	13.00	90.00	7.65	$2.85 \cdot 10^{-4}$	0.43	12.34

Where pH_0 corresponds to the initial pH of the anolyte sample, [Vi] is the anolyte active species concentration, concentration, C.E. is the coulombic efficiency of the first battery cycle, pOH_F and pH_F are the final pOH and pH after the first battery cycle, respectively.

It should be noted that those OH^- generated by the oxygen reduction will over time react with the viologen, resulting in a loss of irreversible capacity in the battery.

Section S2. Synthesis and characterization

Synthesis and Characterization Data of Viologen BSPr-Vi:

Synthesis.

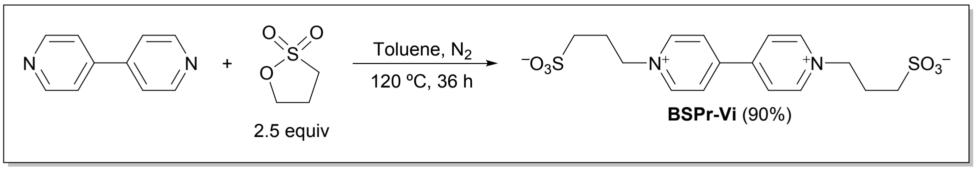

BSPr-Vi was prepared according to a modified procedure of a reported method.[1]

In a round bottom flask (250 mL), 1,3-propanesultone (9.8 g, 80 mmol) was dissolved in anhydrous toluene (60 mL) under an inert nitrogen atmosphere and the obtained solution was heated at 110 ºC. Then, a solution of 4,4'-bipyridine (5 g, 32 mmol) in anhydrous toluene (40 mL) was added slowly with stirring. The reaction mixture was heated at reflux (oil bath, 120 ºC) with vigorous stirring for 36 h. Upon cooling to room temperature, the resulting white precipitate was filtered off, washed several times with hot MeOH and dried under vacuum.

The spectral data of the compound are in good agreement with the reported data.[1]

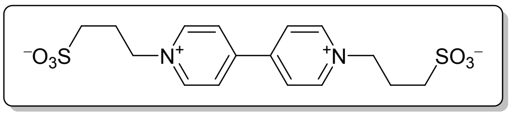

1,1'-Bis(3-sulfonatopropyl)-4,4'-bipyridinium: Yielded (11.22 g, 90%) as a white solid.

^{1}H NMR (300 MHz, D_2O): δ = 9.19 (d, *J* = 6.8 Hz, 4H, Ar*H*), 8.59 (d, *J* = 6.8 Hz, 4H, Ar*H*), 4.92 (t, *J* = 7.4 Hz, 4H, C*H*$_2$), 3.06 (t, *J* = 7.2 Hz, 4H, C*H*$_2$), 2.56 (ap, *J* = 7.3 Hz, 4H, C*H*$_2$).

^{13}C NMR (75.4 MHz, D_2O): δ = 150.4 (2 × C), 145.7 (4 × CH), 127.2 (4 × CH), 60.2 (2 × CH_2), 47.0 (2 × CH_2), 26.2 (2 × CH_2).

^{1}H-NMR spectrum.

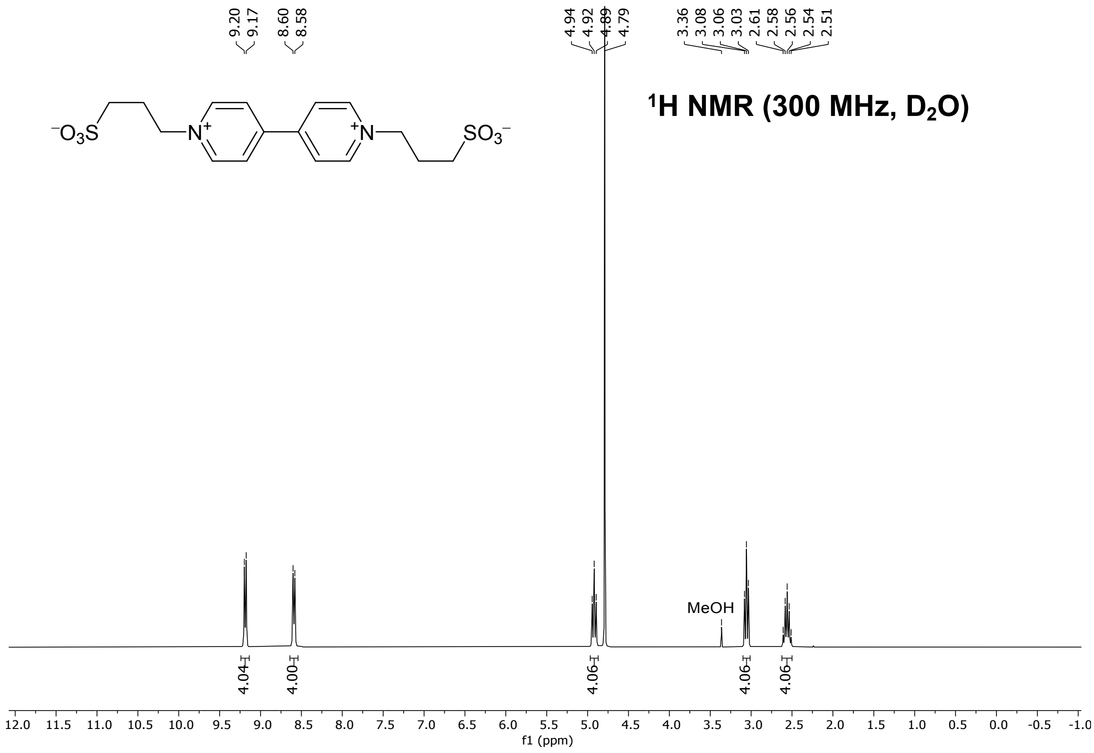

^{13}C-NMR spectrum.

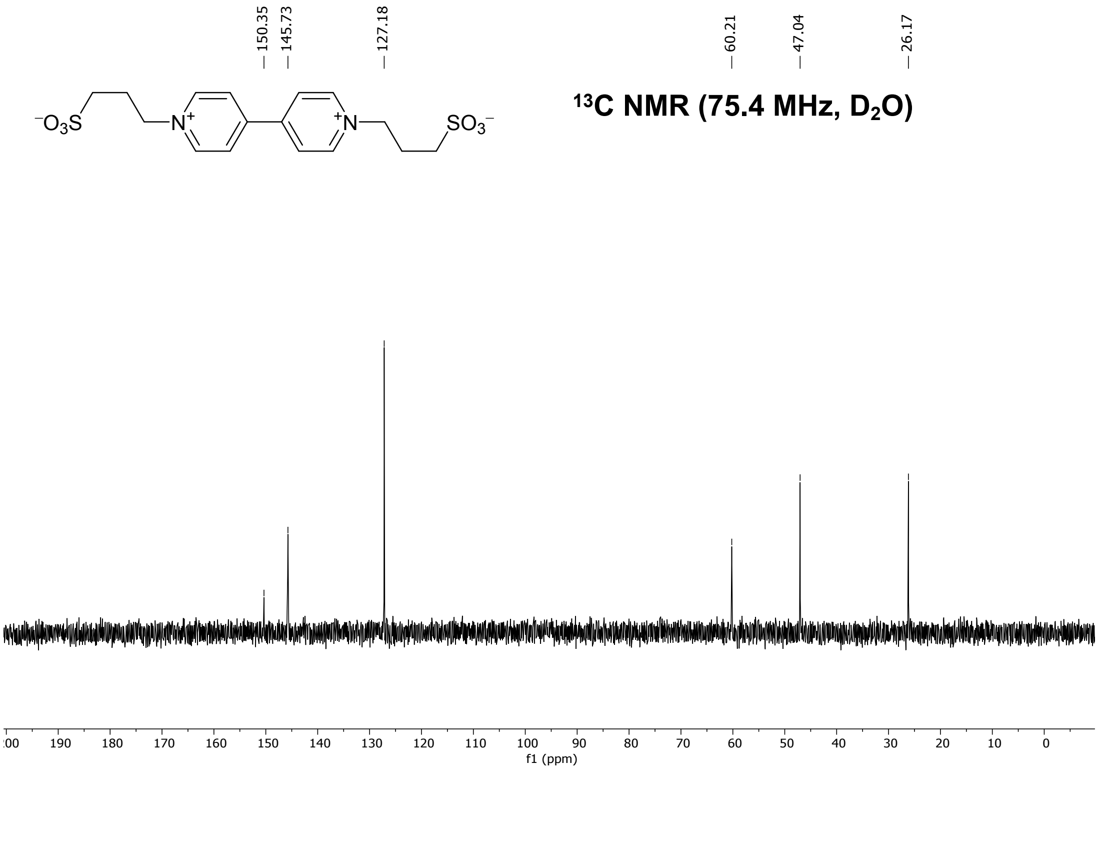

Synthesis and Characterization Data of Compound MSPr:

Synthesis.

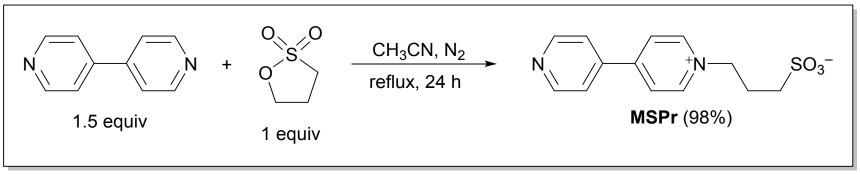

MSPr was prepared according to a modified procedure of a reported method.[2]

In a round bottom flask (50 mL), 4,4'-bipyridine (3 g, 19.2 mmol) was dissolved in anhydrous CH_3CN (35 mL) under an inert nitrogen atmosphere. Then, 1,3-propanesultone (1.55 g, 12.7 mmol) was added with stirring to the obtained solution. The reaction mixture was stirred for 24 h under reflux conditions. Upon cooling to room temperature, the resulting white precipitate was filtered off, washed several times with CH_3CN and dried under vacuum.

The spectral data of the compound are in good agreement with the reported data.[2]

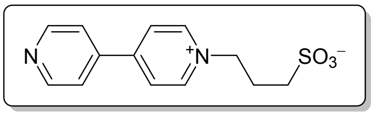

3-([4,4'-Bipyridin]-1-ium-1-yl)propane-1-sulfonate: Yielded (3.45 g, 98%) as a white solid.

^{1}H NMR (300 MHz, D_2O): δ = 9.02 (d, *J* = 6.9 Hz, 2H, Ar*H*), 8.79 (d, *J* = 6.4 Hz, 2H, Ar*H*), 8.44 (d, *J* = 6.8 Hz, 2H, Ar*H*), 7.93 (d, *J* = 6.3 Hz, 2H, Ar*H*), 4.85 (t, *J* = 7.4 Hz, 2H, CH_2), 3.04 (t, *J* = 7.3 Hz, 2H, CH_2), 2.53 (ap, *J* = 7.3 Hz, 2H, CH_2).

^{13}C NMR (75.4 MHz, D_2O): δ = 154.1 (C), 150.0 (2 × CH), 144.9 (2 × CH), 142.5 (C), 126.2 (2 × CH), 122.5 (2 × CH), 59.8 (CH_2), 47.0 (CH_2), 26.1 (CH_2).

¹H-NMR spectrum.

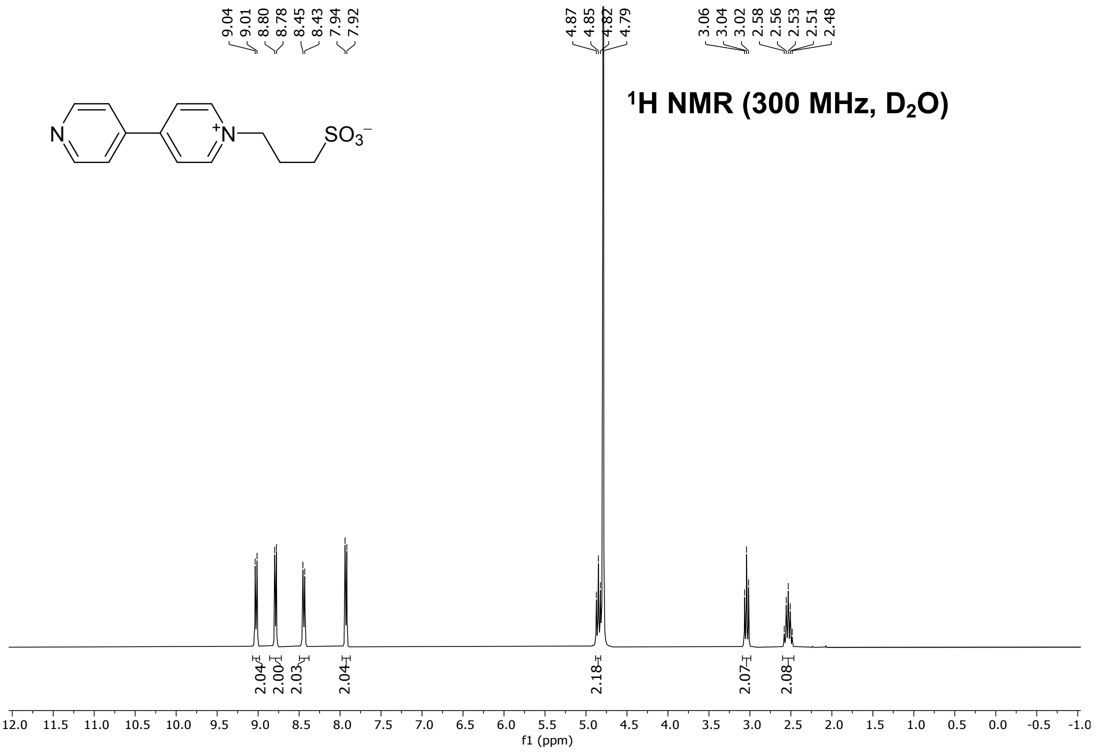

¹³C-NMR spectrum.

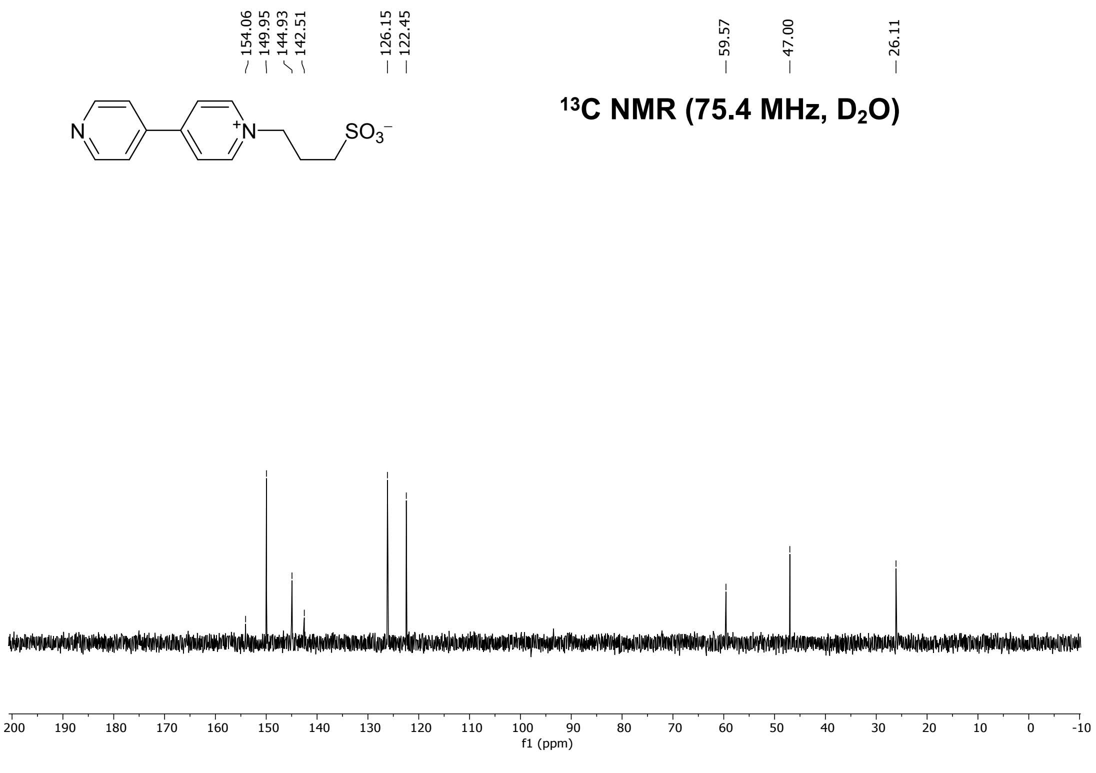

Synthesis and Characterization Data of 1,3-Butanesultone:

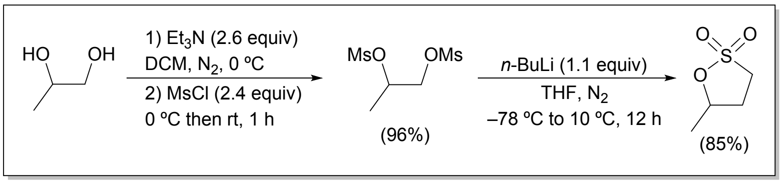

1,3-Butanesultone was prepared in two steps from propylene glycol according to modified procedures of reported methods.[3]

Synthesis.

Procedure for the synthesis of propane-1,2-diyl dimethanesulfonate: In an oven dried Schlenck flask (250 mL), propylene glycol (4.56 g, 60 mmol) and anhydrous DCM (150 mL) were added under an inert nitrogen atmosphere. The resulting solution was cooled to 0 ºC and Et_3N (21.9 mL, 156 mmol) was added. Then, methanesulfonyl chloride (MsCl; 11.1 mL, 144 mmol) was added slowly via a syringe under vigorous stirring and the mixture was stirred at 0 ºC for 1 h. The obtained suspension was allowed to warm and stirred at room temperature for 1 h. The reaction was quenched with water (100 mL). The layers were separated and the aqueous layer was extracted with DCM (2 × 75 mL). The combined organic layers were washed with water and brine, dried over anhydrous Na_2SO_4, filtered and concentrated under reduced pressure. The product was obtained pure and used in the next step without need for further purification. The product is purified by crystallization (toluene/hexane), if required.

The spectral data of the compound are in good agreement with the reported data.[4]

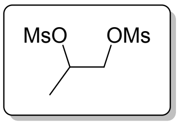

Propane-1,2-diyl dimethanesulfonate: Yielded (13.38 g, 96%) as a yellowish solid.

^{1}H NMR (300 MHz, $CDCl_3$): δ = 4.99 (pd, *J* = 6.6 and 3.1 Hz, 1H, C*H*), 4.32 (dd, *J* = 11.6 and 3.1 Hz, 1H, CH*H*), 4.22 (dd, *J* = 11.6 and 6.7 Hz, 1H, C*H*H), 3.07 (s, 3H, C*H*$_3$), 3.06 (s, 3H, C*H*$_3$), 1.46 (d, *J* = 6.6 Hz, 1H, C*H*$_3$).

^{13}C NMR (75.4 MHz, $CDCl_3$): δ = 75.8 (CH), 70.5 (CH_2), 38.8 (CH_3), 37.9 (CH_3), 17.5 (CH_3).

LR-MS (EI), *m/z* (%): 158 (25), 123 (100), 79 (88).

^{1}H-NMR spectrum.

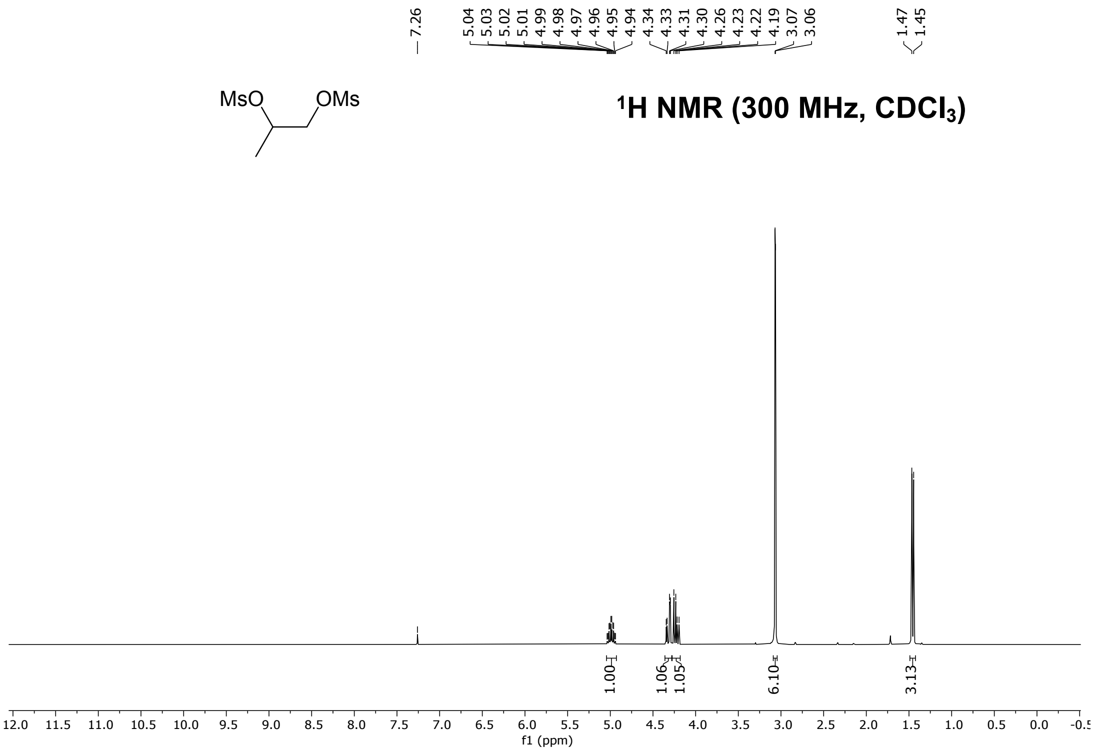

^{13}C-NMR spectrum.

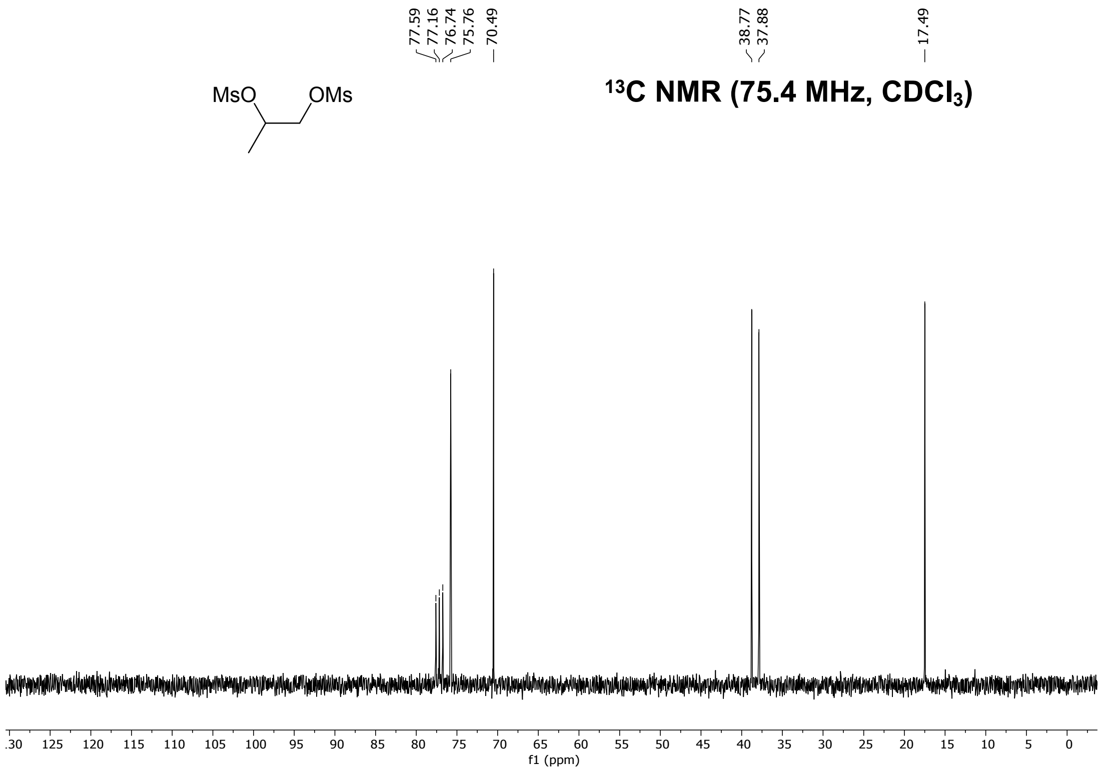

Procedure for the synthesis of 1,3-Butanesultone: In an oven dried Schlenck flask (250 mL), propane-1,2-diyl dimethanesulfonate (11.6 g, 50 mmol) and anhydrous THF (150 mL) were added under an inert nitrogen atmosphere. The resulting solution was cooled to −78 ºC and *n*-BuLi (22 mL of a 2.5 M solution in hexanes, 55 mmol) was added under vigorous stirring. Then, the obtained suspension was stirred at 10 ºC for 12 h. The reaction was quenched with water (100 mL) and the reaction mixture was extracted with DCM (3 × 70 mL). The combined organic layers were washed with brine, dried over anhydrous Na_2SO_4, filtered and concentrated under reduced pressure giving rise to the desired product. The sultone was obtained in pure form and used without further purification in the next step. Alternatively, the addition of *n*-BuLi can be carried out at higher temperature, although a lower yield was obtained and the crude product should be further purificated.

The spectral data of the compound are in good agreement with the reported data.[5]

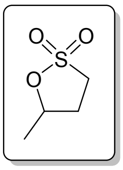

1,3-Butanesultone: Yielded (5.8 g, 85%) as a yellowish oil.

^{1}H NMR (300 MHz, $CDCl_3$): δ = 4.78 (dq, *J* = 8.7 and 6.1 Hz, 1H, C*H*), 3.40–3.32 (m, 1H, CH*H*), 3.27 (ddd, *J* = 13.1, 9.3 and 7.9 Hz, 1H, C*H*H), 2.63 (dddd, *J* = 13.5, 7.9, 5.9 and 4.4 Hz, 1H, CH*H*), 2.28 (dq, *J* = 13.1 and 9.0 Hz, 1H, C*H*H), 1.53 (d, *J* = 6.1 Hz, 3H, CH_3).

^{13}C NMR (75.4 MHz, $CDCl_3$): δ = 79.2 (CH), 46.1 (CH_2), 31.3 (CH_2), 20.9 (CH_3).

LR-MS (EI), *m/z* (%): 121 (100), 43 (81), 29 (42).

^{1}H-NMR spectrum.

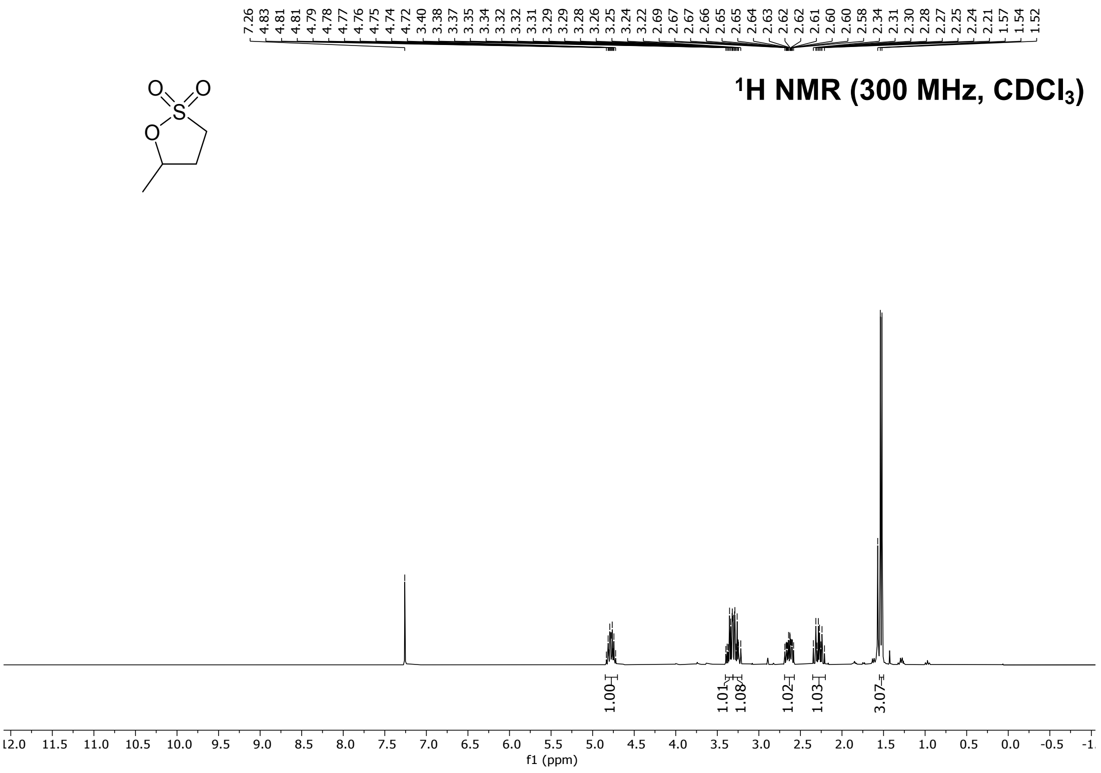

^{13}C-NMR spectrum.

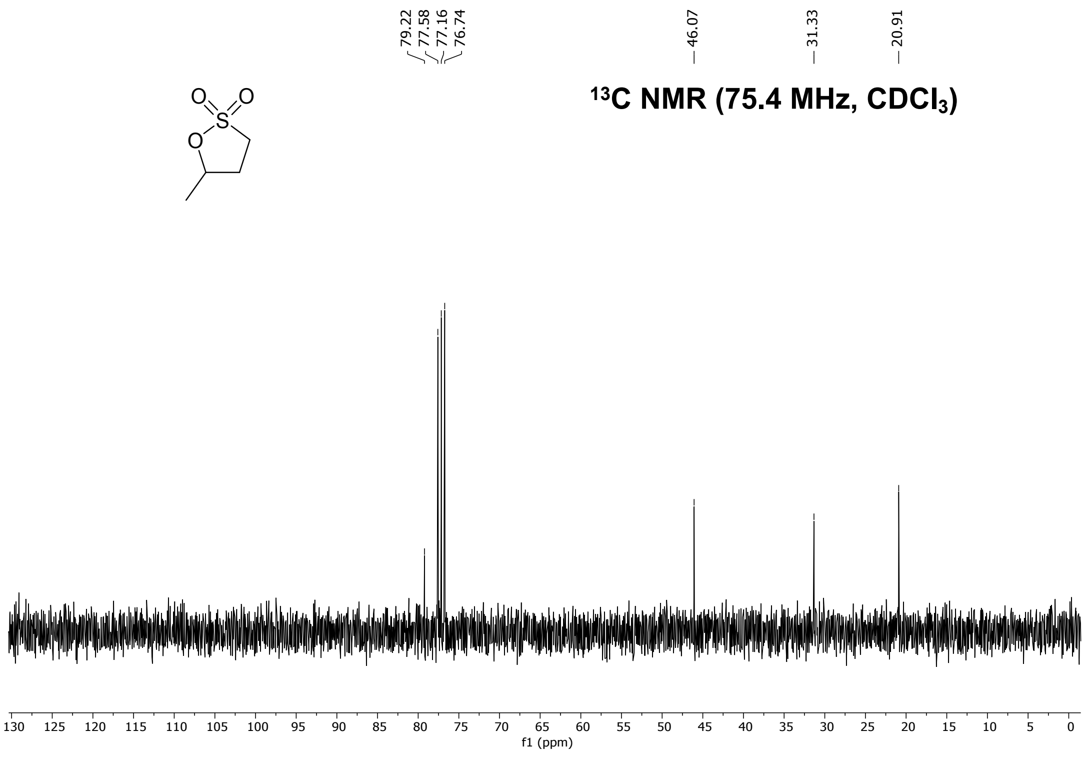

Synthesis and Characterization Data of Viologen BS3Bu-Vi:

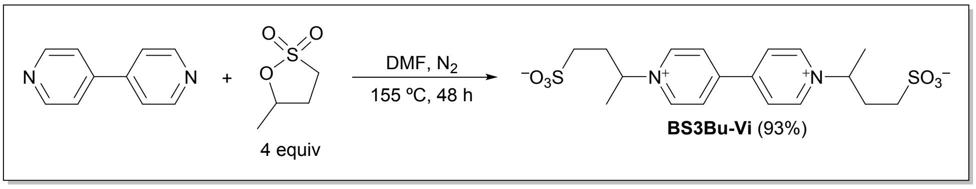

Synthesis.

In a screw-cap Schlenk flask (48 mL), 1,3-butanesultone (2.18 g, 16 mmol) was dissolved in anhydrous DMF (25 mL) under an inert nitrogen atmosphere. Then, 4,4'-bipyridine (625 mg, 4 mmol) was added with stirring to the resulting solution. The Schlenk flask was sealed and the reaction mixture was heated at 155 ºC with vigorous stirring for 48 h. Upon cooling to room temperature, the resulting white precipitate was filtered off, washed several times with MeOH and dried under vacuum.

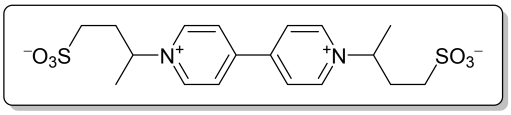

1,1'-Bis(1-methyl-3-sulfonatopropyl)-4,4'-bipyridinium: Yielded (1.59 g, 93%) as a white solid, Mp (from MeOH) 296–298 ºC (decomp.).

^{1}H NMR (300 MHz, D_2O): δ = 9.23 (d, *J* = 6.7 Hz, 4H, Ar*H*), 8.61 (d, *J* = 6.7 Hz, 4H, Ar*H*), 5.20 (h, *J* = 7.0 Hz, 2H, C*H*), 3.02–2.81 (m, 4H, CH_2), 2.55 (q, *J* = 7.3 Hz, 4H, CH_2), 1.82 (d, *J* = 6.7 Hz, 6H, CH_3).

^{13}C NMR (75.4 MHz, D_2O): δ = 151.1 (2 × C), 144.8 (4 × CH), 128.0 (4 × CH), 68.7 (2 × CH), 47.4 (2 × CH_2), 32.4 (2 × CH_2), 21.0 (2 × CH_3).

HR-MS (ESI+) *m/z*: $[M+H]^+$ calcd. for $C_{18}H_{25}N_2O_6S_2$, 429.1149; found, 429.1153.

^{1}H-NMR spectrum.

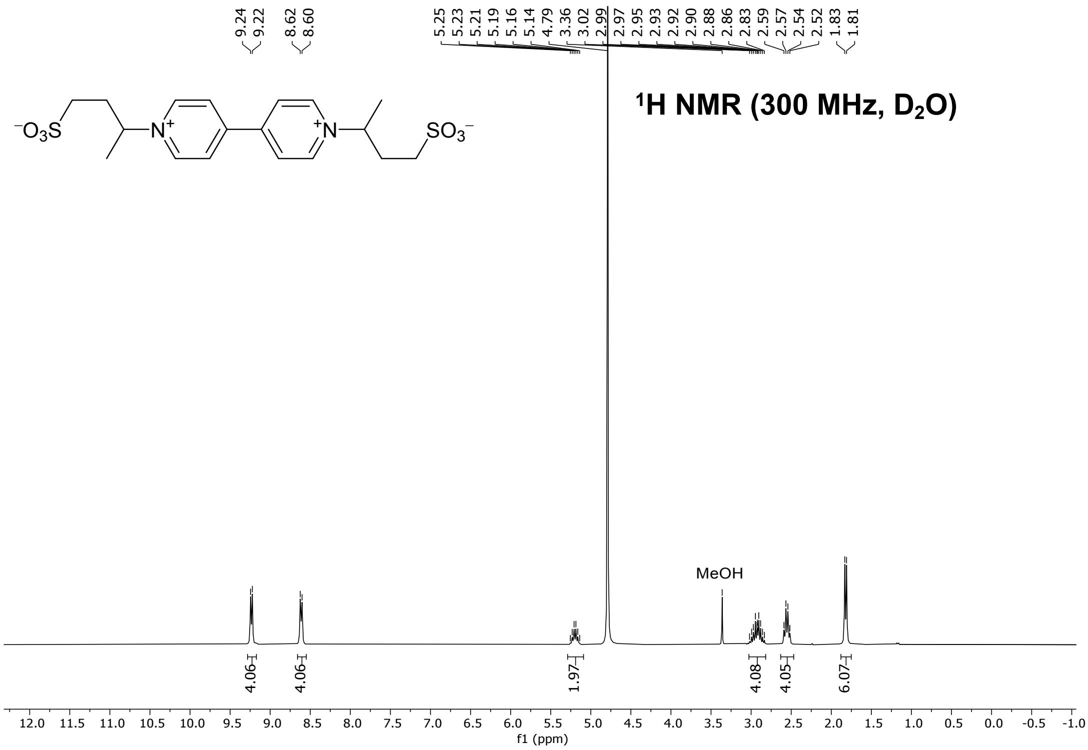

^{13}C-NMR spectrum.

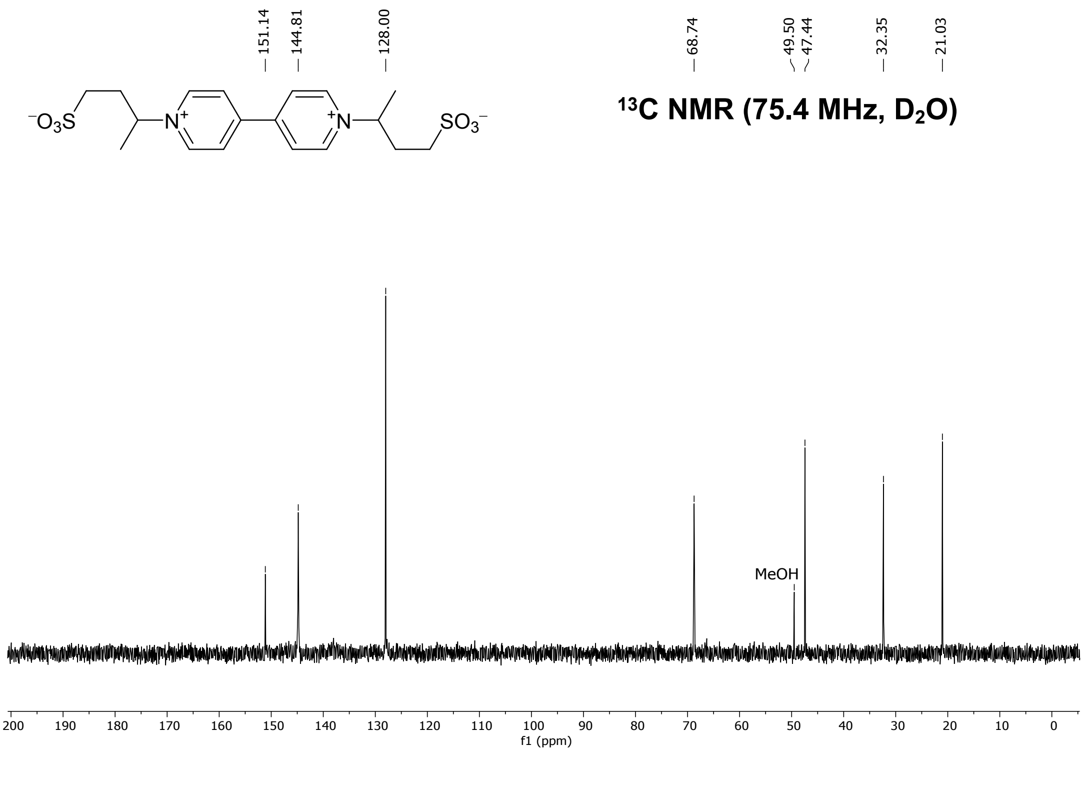

Electrochemical characterization.

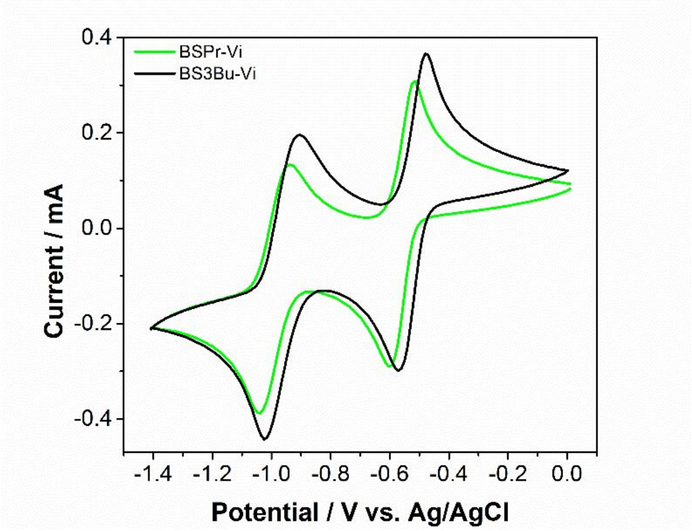

Figure S1. CV curves of 25 mM **BSPr-Vi** (green) and **BS3Bu-Vi** (black) in 1 M KCl with a scan ratee of 100 mVs^{-1}, respectively.

Synthesis and Characterization Data of Compound MS3Bu:

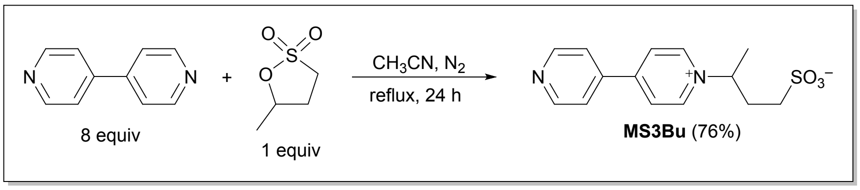

Synthesis.

In a round bottom flask (50 mL), 4,4'-bipyridine (3.8 g, 24.3 mmol) was dissolved in anhydrous CH_3CN (25 mL) under an inert nitrogen atmosphere. Then, 1,3-butanesultone (420 mg, 3.1 mmol) was added with stirring to the obtained solution. The reaction mixture was heated at reflux with stirring for 36 h. Upon cooling to room temperature, the resulting white precipitate was filtered off, washed several times with Et_2O and dried under vacuum.

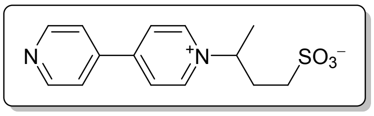

3-([4,4'-Bipyridin]-1-ium-1-yl)butane-1-sulfonate: Yielded (684 mg, 76%) as a white solid, Mp 260–262 ºC (decomp.).

^{1}H NMR (300 MHz, D_2O): δ = 9.07 (d, *J* = 6.8 Hz, 2H, Ar*H*), 8.77 (d, *J* = 6.2 Hz, 2H, Ar*H*), 8.45 (d, *J* = 6.7 Hz, 2H, Ar*H*), 7.92 (d, *J* = 6.2 Hz, 2H, Ar*H*), 5.12 (h, *J* = 6.8 Hz, 1H, C*H*), 3.01–2.79 (m, 2H, CH_2), 2.52 (q, *J* = 7.4 Hz, 2H, CH_2), 1.79 (d, *J* = 6.7 Hz, 3H, CH_3).

^{13}C NMR (75.4 MHz, D_2O): δ = 154.3 (C), 150.0 (2 × CH), 143.4 (2 × CH), 142.5 (C), 126.4 (2 × CH), 122.5 (2 × CH), 67.3 (CH), 46.8 (CH_2), 31.7 (CH_2), 20.4 (CH_3).

HR-MS (ESI+) *m/z*: $[M+H]^+$ calcd. for $C_{14}H_{17}N_2O_3S$, 293.0954; found, 293.0957.

^{1}H-NMR spectrum.

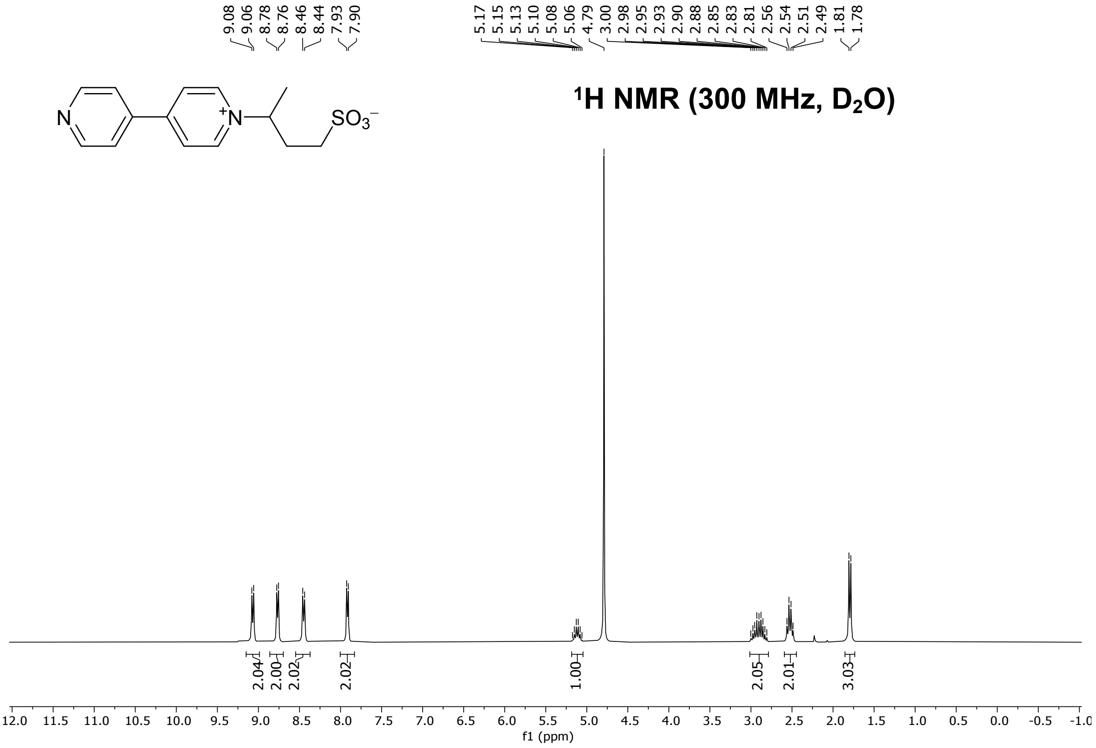

^{13}C-NMR spectrum.

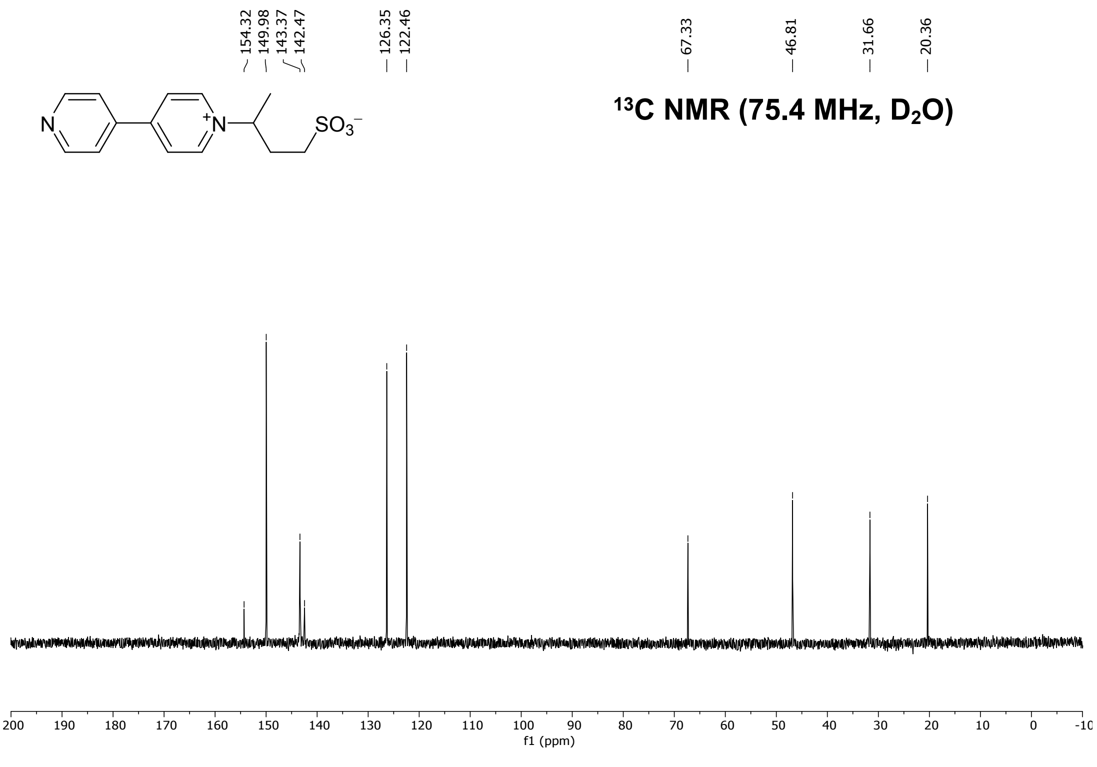

Section S3. Comparative capacity retention

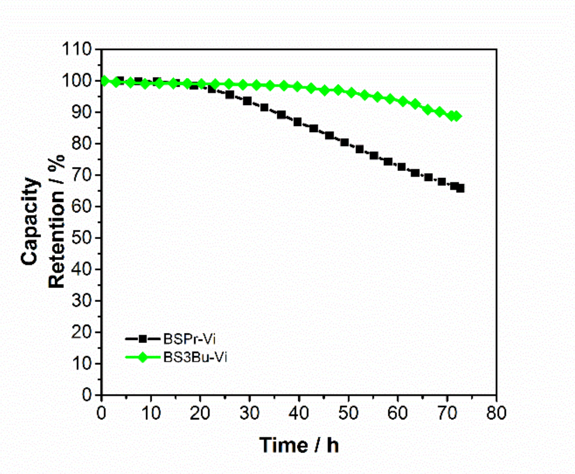

Figure S2. Evolution of the charge capacity upon cycling over 72 h for the **BSPr-Vi** // $K_4[Fe(CN)_6]$ battery (green) and the **BS3Bu-Vi** // $K_4[Fe(CN)_6]$ battery (black).

Section S4. Anolytes crossover evaluation

We attribute the total capacity loss to chemical degradation and not to anolyte crossover. The contribution of the anolyte crossover to the capacity fading was negligible as we have estimated *via* CV post mortem analysis of the catholytes for both batteries (Fig. S4).

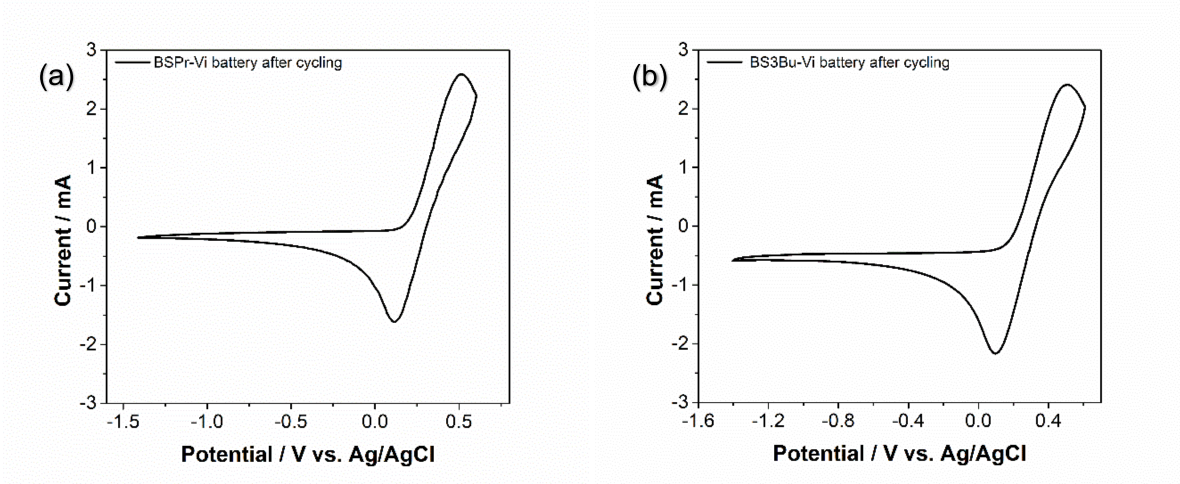

Figure S3. CV post mortem analysis of the catholyte for (a) **BSPr-Vi** // $K_4[Fe(CN)_6]$ and (b) **BS3Bu-Vi** // $K_4[Fe(CN)_6]$ batteries.

Section S5. Capacity fading and NMR post mortem analysis for BS3Bu-Vi

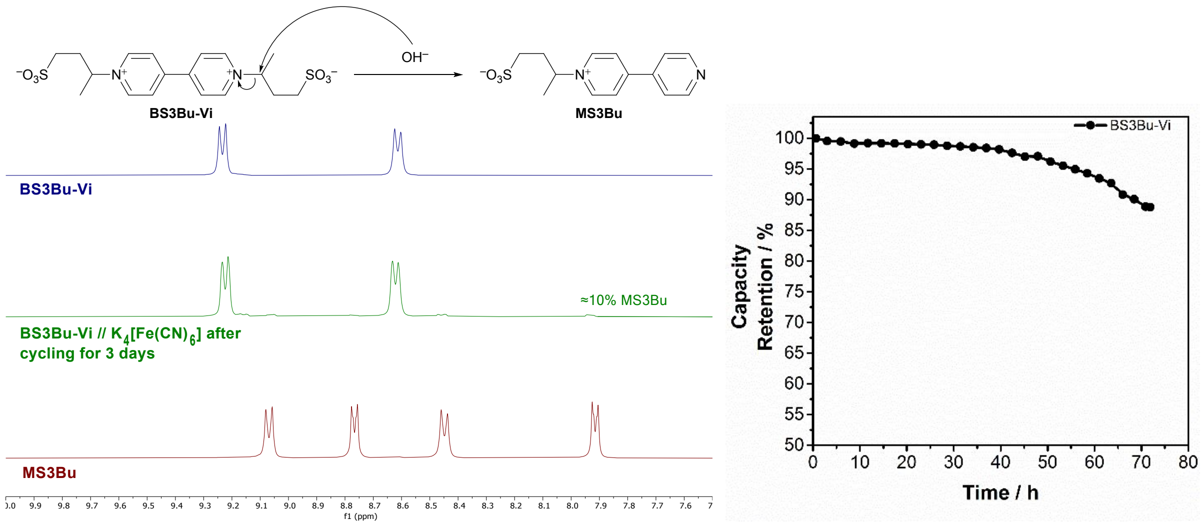

Figure S4. ^{1}H NMR post mortem analysis of the **BS3Bu-Vi** // $K_4[Fe(CN)_6]$ battery anolyte after 3 days and capacity fading of the **BS3Bu-Vi** // $K_4[Fe(CN)_6]$ battery during 3 days.

References

1. J. Luo, B. Hu, C. Debruler, Y. Bi, Y. Zhao, B. Yuan, M. Hu, W. Wu and T. L. Liu, *Joule*, 2019, **3**, 149–163.
2. C. DeBruler, B. Hu, J. Moss, X. Liu, J. Luo, Y. Sun and T. L. Liu, *Chem*, 2017, **3**, 961–978.
3. a) T. Harada, T. Mai, T. Tuyet and A. Oku, *Org. Lett.*, 2000, **2**, 1319–1322. b) T. Durst and K.-C. Tin, *Can. J. Chem.*, 1970, **48**, 845–851.
4. N. Ortega, A. Feher-Voelger, M. Brovetto, J. I. Padrón, V. S. Martín and T. Martín, *Adv. Synth. Catal.*, 2011, **353**, 963–972.
5. G. M. T. Smith, P. M. Burton and C. D. Bray, *Angew. Chem. - Int. Ed.*, 2015, **54**, 15236–15240.
